# School policies and practices associated with Thai children’s overall and domain specific physical activity

**DOI:** 10.1371/journal.pone.0245906

**Published:** 2021-01-22

**Authors:** Areekul Amornsriwatanakul, Leanne Lester, Michael Rosenberg, Fiona Bull

**Affiliations:** 1 College of Sports Science and Technology, Mahidol University, Phutthamonthon, Nakhon Pathom, Thailand; 2 School of Human Sciences (Exercise and Sport Science), University of Western Australia, Nedlands, Perth, Australia; 3 Centre for Built Environment and Health, School of Human Sciences, University of Western Australia, Nedlands, Perth, Australia; La Inmaculada Teacher Training Centre (University of Granada), SPAIN

## Abstract

School has a significant role in providing opportunities for children to engage in physical activity (PA) through policies and practices. This study aimed to identify the prevalence of school policies and practices related to physical activity (PA) and their association with Thai students’ overall and domain specific PA. This cross-sectional analysis included 5,830 students aged 6–17 years from 136 schools recruited though a multi-stage stratified cluster sampling across Thailand. Student’s PA data were assessed using a student survey and school data were collected by a principal survey. Associations between students’ PA and school variables were examined using logistic regressions. Despite a high prevalence of Thai schools reporting many policies and practices promoting PA in different areas, students reported low levels of PA. None of the school PA policies and practices was associated with students’ overall and domain specific activities, with active transport as the one exception. When schools had an active transport policy, students were 40% (OR = 1.40, p = 0.01) more likely to travel actively to/from school. The identified positive relationship between school active transport policy and students’ active travel behavior suggests a potential wider adoption of the policy promoting school active transport aiming to increase student’s PA levels among all Thai schools. Intervention studies are necessary to confirm this finding. Our study also reflected that, for greater levels of children’s school-based PA, strategies to translate the existing school PA policies into effective implementation should be an emphasis for Thai schools.

## Introduction

Worldwide, overall levels of physical activity (PA) among school-age children are low [1]. There have been international and national efforts to advocate for higher PA participation in this population group, and school environment has been highlighted. A Global Action Plan on Physical Activity, launched by the World Health Organization (WHO) in 2018, recommended that member states should ensure provision of opportunities for PA at education institutions [2]. From a public health perspective, school is an ideal setting where a majority of children spend a substantial amount of their weekday time and can therefore be reached regardless of their race and religion. School is a strategic setting not only for the provision or promotion of PA at the population level, but also for increasing and sustaining it. With regard to this significant role of school, the school environment, i.e. policies and practices, can potentially influence PA behaviors in school-age children [3].

Policy refers to a formal written statement of intent that defines priority and the parameters for decisions and actions required to achieve desirable outcomes. A school policy can be mandated at different levels from national, state/regional, district, and the school itself [3]. Once established and implemented, policies should shape school practices and delivery of PA programs in a sustainable manner. PA programs can be provided through either specific PA policies or holistic health promoting policies. Specific PA policies involve a provision of opportunities for students to be physically active commonly through physical education (PE), recess, extracurricular activities or activities outside school hours, active transport to/from school (i.e., walk or bike), and provision of school PA facilities [4]. PA programs can also be provided through a holistic health promoting policy; e.g., WHO Health Promoting School (HPS) framework which incorporates health policy as an integral element [5].

Overall, review of current evidence suggests that the association between school PA policy and students’ participation in PA is complex, and the knowledge base in this area remains incomplete [3]. Available evidence mostly conducted in developed countries suggests that some specific policies, such as a provision of PE class [6], and recess [7] have no impacts on student’s overall PA levels. However, other specific policies seem to positively influence students’ participation in PA. For example, when schools have policies to provide extracurricular activities for students to be physically active, there appears to be positive impacts on students’ PA levels [6,8]. Nonetheless, evidence on contributions of these policies on children’s overall and particularly domain specific PA is scarce and warrants further investigation.

School practices are referred to decisions and actions guided by policies, and in this case, related to a provision of school PA facilities and programs [9]. The practices differ greatly between schools as they are multifaceted and involve multiple concerned parties. Frequency and duration of PE class and a provision of PE specialists were frequently examined as concrete practices following adopted PE policies. Previous evidence showed that the frequency and duration of PE class consistently had no association with students’ PA [6], whereas a provision of a PE specialist showed a positive association with class time moderate to vigorous PA [10]. The delivery of PE class in a majority of countries have legal requirements for the implementation and consequently all schools across the country have to comply [11]. In Thailand, PE is bundled with health education (HE) and is compulsory as it is mandated by the Ministry of Education. The national education curriculum clearly specifies the maximum class time allocation for PE/HE per academic year (i.e., 80 hrs for grades 1–9, and 120 hrs for grades 10–12) [12]. In practice, Thai schools typically provide <60 minutes of PE/week.

Activities during PE class and active play have received significant recognition from international agencies. PE has been highlighted in the Kazan Action Plan of UNESCO as the most important means to ensure lifelong participation in PA [13], while active play is the right of the child and necessary for their healthy growth and development [14]. Active play (defined as spontaneous, freely chosen, self-directed, unstructured, and unorganized [15]) is a relatively new area of study and active play policies are rarely examined independently [16]. They are typically investigated indirectly through policies providing time for free play [17], recess [18], and non-curricular activities [7]. Moreover, active play has continuously been identified as an alarming issue by a regular national assessment and raised as a national campaign in Thailand since 2016 [19]. Active transport has been promoted as a potential means for increasing PA in people’s daily life, and offers promising opportunities for countries to concurrently achieve many sustainable development goals [2]. Schools that have a policy to promote active transport to/from school report a larger proportion of students who travel actively and a higher number of active trips made by students, as well as a larger proportion of students who meet PA guidelines [20–22].

International literature on the prevalence of school PA policies and their influence on children’ PA levels is growing gradually. However, there is a dearth of evidence from less developed countries. The body of knowledge is lacking particularly in the contributions of school PA policies and practices on children’ domain specific activity. Importantly, it is timely for Thailand to obtain baseline insights into school PA contexts as “Active Children” was one of the three policies recently adopted by the National Committee of the National Action Plan on PA [23]. This present study investigates the prevalence of school policies and practices related to PA and their association with Thai children’s overall and domain specific PA. The information is timely to help plan for national school-related policies in Thailand and will be beneficial for other countries.

## Methods

### Participants

In this study, 5,830 students aged 6–17 years, and 136 school principals or nominated representatives from Thai primary and secondary schools participated in the “Thailand Physical Activity Children Survey (TPACS) 2015”. TPACS is a cross-sectional population survey conducted in 16,788 children and adolescents aged 6–17 years. They were recruited through a multi-stage stratified clustered sampling from 336 schools in 27 provinces across 9 regions in Thailand including Bangkok Metropolitan area. Full details about TPACS and the sampling conducted at each stage for both students and school principals are provided in detail elsewhere [19].

### Measures

TPACS Student Questionnaire (TPACS-SQ) and TPACS School Principal Questionnaire (TPACS-SPQ) were used in this study [24]. Further details on development of the TPACS-SQ and its validity and reliability are provided elsewhere [19].

To assess children’s ***overall PA levels*** according to WHO PA guidelines [25], students were asked the number of days they were active for a combined total of at least 60 min/day over the past 7 days. Students who were active ≥60 min/day on all days were classified into “Meeting PA guidelines” and ≤6 days into “Not meeting PA guidelines”. Since ***participation in PE*** is compulsory in Thailand, students were asked to ensure if they had any PE class during the current semester and responses were categorized into “Yes” or “No”. ***Active play*** was assessed by asking what kind of activities students did during four free school periods (before and after school, lunch, and breaks between classes) by providing a list of potential activities. Inactive activities were excluded before computing how many free periods students were active and classified into “<2 free periods” and “≥2 free periods”. ***Active travel*** *to/from school* was assessed by asking how children usually travel to/from school and then classifying travel into either “active” or “inactive”.

The **TPACS-SPQ** assessed school policies and practices related to PA promotion. Based on items selected from previously validated questionnaires [26–28], items from these questionnaires were adjusted slightly to suit the Thai education system and school context (e.g., school governing system). The TPACS-SPQ was translated into Thai for face validity testing and data collection. After pilot testing the draft questionnaire with three school principals for comprehension and face validity, the principals provided qualitative feedback to improve the final questionnaire. The final TPACS-SPQ had a total of 22 items (some were multi-items) comprising four major sections: 1) school characteristics, 2) school PA policy and programs such as PE and HPS, 3) socio-cultural characteristics, and 4) principal’s individual information.

To assess if schools had any ***policies*** promoting PA in place, a list of policies including active play, active transport (i.e., bike or walk to/from school), extracurricular activity (e.g., intramural or interscholastic sport competitions), provision of PA facilities, student access to PA facilities outside school hours, shared use of facilities with the community, and HPS, was provided with four response options (“Yes”/“No”/“Under development”/“Don’t know or Not sure”). “Under development” responses were collapsed into “No”. School principals were also questioned if their school had any other policies related to the promotion of PA, sports, exercise and recreation which was not present in the list. The same response options were applied. Recess policy was assessed separately because further information concerning frequency and duration were also asked.

School ***practices*** concerning the provision of PE class were assessed by asking about ***PE class time/week*** (min.), and if there were any ***PE specialists*** graduated with specialist skills (“Yes”/”No”) delivering the PE class. School principals were questioned if the school organized any ***extracurricular activities*** which provided students with opportunities to be physically active outside school hours on school days, excluding PE (“Yes”/“No”). ***Student access to in/outdoor PA facilities outside school hours*** were assessed by asking if school allowed students to use in/outdoor PA facilities before and after school (“Yes”/“No”/“Don’t know/Not sure”). ***Community access to in/outdoor PA facilities outside school hours*** were assessed in a similar fashion.

### Procedure

Trained research staff administered TPACS-SQ using a data collection protocol which was developed for different age groups to accommodate the differences in student capability and maturity. For TPACS-SPQ, school principals were recruited to participate by mail. Full details concerning data collection protocols of both questionnaires is available elsewhere [19]. Data of both surveys were entered twice, checked against hard copies to rectify discrepancies, with final datasets from each area centrally collated and systematically cleaned. Student and principal survey data collected across June 2015–January 2016 were merged and thoroughly checked for eligibility and cases with missing values. Eventually, a total of 5,830 students clustered into 136 of 149 schools (91%) remained for the analysis and were weighted against age, sex, and regional distributions provided by the Ministry of Education [29]. Study procedures were approved by the Institution for the Development of Human Research Protections in Thailand and the Human Research Ethics Committee of The University of Western Australia (RA/4/1/7335). The study adopted active school and passive parental consent, both of which were approved by both Institutional Review Boards. Because schools and school principals are considered guardians of students, the recruitment method used was the active school participation, which is commonly used in Thailand for research in children. After the school principal approved of and provided written consent for the study, it was then conducted.

### Data analysis

Descriptive statistics were used to describe sample characteristics and prevalence of participation in PA. Differences between proportions of the samples in each category of exploratory categorical variables were examined by Chi-square tests (t-tests for continuous variables). Multiple logistic regressions taking into account school clustering effects were performed to examine an association between explanatory and outcome variables. The analyses were performed separately for overall PA levels, and domain specific activities. Inclusion of explanatory variables in each regression model was guided by the literature. Potential confounders including sex, age, BMI, and region were selected based on previous research [30], and were accounted for in the multivariable regressions. The descriptive statistics and Chi-square or t-tests were performed in SPSS v26, and all regressions were conducted in STATA v15.

## Results

Demographic data and descriptive statistics of the student samples are provided in Table 1. The proportions of students that participated in this study by sex and age categories were almost equal, and almost 30% of them resided in the north of Thailand. Half the students had normal weight (50.3%) although less than one quarter of students (21%) met the current global PA guidelines, and only 18% of students reported active play in ≥2 free periods at school across a school week. Almost all students (92.4%) reported PE classes in the current semester, and just over half (52.6%) traveled actively to/from school.

**Table 1:** Descriptive information of the student samples.

Variable	n (%)
***Sex***	
Boys	3013 (51.7)
Girls	2817 (48.3)
***Age***	
6–9 years old	1979 (33.9)
10–13 years old	1907 (32.7)
14–17 years old	1944 (33.3)
***BMI***	
Underweight	879 (15.1)
Normal	2935 (50.3)
Overweight	1008 (17.3)
Obese	1008 (17.3)
***Religion***	
Buddhism	5470 (93.8)
Islamic	287 (4.9)
Christian	73 (1.3)
***Geographical region***	
Bangkok	454 (7.8)
East	870 (14.9)
West	314 (5.4)
North	1701 (29.2)
South	1084 (18.6)
North East	1100 (18.9)
Central	307 (5.3)
***Participation in PA***	
Overall PA levels	1218 (20.9)
Physical education class	5393 (92.4)
Active play (≥2 free period)	1042 (17.9)
Active travel to/from school	3068 (52.6)

The prevalence of different school policies and practices promoting PA are shown in Table 2. All schools reported a PE policy, whereas only one third of schools reported a policy on recess (35.3%). Most schools (87.5%) provided <60 min of PE class/week and a high proportion of schools (77.2%–94.1%) reported many practices supporting PA promotion. Table 2 also presents results from univariable regressions of school policies and practices potentially associated with students’ overall PA levels. The policy on shared use of school PA facilities with the community had a slightly inverse relationship with students’ overall PA levels (OR = 0.75, 95%CI: 0.56–1.0, p = 0.048). However, after accounting for sex, age, BMI, region, and other school variables in the multiple regressions, no school variables had a significant relationship with students’ overall PA levels.

**Table 2:** Univariable regression results of school variables associated with students’ meeting PA guidelines (n school = 136, n student = 5,830)^†^.

	No		Yes		OR	(95%CI)	p
	% School prevalence	% Student meeting PA guideline	% School prevalence	% Student meeting PA guideline			
**Policy**							
Physical education	0.0	N/A	100.0	20.9	N/A		
Recess	64.7	21.5	35.3	20.0	0.91	(0.72, 1.16)	0.454
Extracurricular activities	1.5	N/A	98.5	20.8	N/A		
PA facilities/equipment	6.6	18.4	93.4	21.0	1.17	(0.96, 1.43)	0.110
Students access to PA facilities/Equipment outside school hours	16.9	20.2	83.1	21.0	1.05	(0.80, 1.39)	0.719
Shared use of facilities with community	11.0	25.5	89.0	20.4	0.75	(0.56, 1.00)	0.048*
Involvement in HPS	12.6	19.5	87.4	21.2	1.11	(0.82, 1.50)	0.488
Active play	47.8	20.2	52.2	21.5	1.08	(0.87, 1.35)	0.490
Active transport	50.7	20.7	49.3	21.0	1.02	(0.81, 1.28)	0.880
Other PA-related policies	7.0	22.1	93.0	20.8	0.93	(0.76, 1.13)	0.461
**Practice**							
PE class time: >60min/week	87.5	21.3	12.5	18.4	0.83	(0.64, 1.09)	0.184
Having PE specialists	22.1	23.4	77. 9	20.4	0.84	(0.62, 1.14)	0.259
Annual fitness test	7.4	23.5	92.6	20.7	0.85	(0.64, 1.11)	0.223
Organization of ECA	5.9	20.9	94.1	20.9	0.99	(0.68, 1.43)	0.955
Student access to indoor PA facilities outside school hours	19.1	20.3	80.9	21.0	1.04	(0.78, 1.39)	0.797
Student access to outdoor PA facilities outside school hours	6.6	22.6	93.4	20.7	0.89	(0.62, 1.29)	0.534
Community access to indoor PA facilities outside school hours	44.1	21.4	55.9	20.5	0.94	(0.76, 1.18)	0.619
Community access to outdoor PA facilities outside school hours	22.8	20.4	77.2	21.1	1.05	(0.86, 1.31)	0.706
Parents invited to join activities	82.5	23.0	17.5	20.3	0.85	(0.67, 1.08)	0.180
**School characteristics**							
Setting	**City district**		**Not city district**				
	55.9	21.0	44.1	20.7	0.99	(0.80, 1.21)	0.895
Neighborhood issues	**Low**		**High**				
	80.1	21.3	19.9	19.2	0.88	(0.70, 1.09)	0.255

*p<0.05, OR: Odds Ratio, CI: Confidence Interval, Ref: Reference category, N/A: Not available due to insufficient variation for calculation, PA: Physical activity, HPS: Health promoting school, PE: Physical education.

^†^Results of multivariable regressions are not shown as no significant associations were found between explanatory and outcome variables.

Table 3 shows regression results of school policies and practices associated with students’ participation in PE class. The multivariable regression results revealed that students were almost three times more likely (OR = 3.54, 95%CI: 1.39–9.01, p = 0.008) to participate in PE class when schools reported other policies related to PA promotion, compared to those from schools that reported no other policies.

**Table 3:** Regression results of school variables associated with students’ participation in PE class.

	% Participation in PE class		Univariable regression			Multivariable regression^a^		
	No (Ref)	Yes	OR	(95%CI)	P	OR	(95%CI)	P
**Policy**								
PA facilities/equipment	94.4	92.3	0.69	(0.22, 2.21)	0.538			
Other PA-related policies	81.2	93.2	3.16	(0.88, 11.45)	0.079	3.54	(1.39, 9.01)	0.008†
**Practice**								
PE class time: >60min/week	93.3	87.0	0.48	(0.17, 1.32)	0.156			
Have PE specialist (Ref: No)	88.8	93.2	1.72	(0.68, 4.36)	0.250			
Organization of ECA (Ref: No)	96.9	92.3	0.36	(0.11, 1.16)	0.088			
Annual fitness test	90.4	92.6	1.31	(0.43, 3.96)	0.628			
**Characteristic**	**City district (Ref)**	**Non-city district**						
School setting	94.1	89.6	0.54	(0.25, 1.13)	0.103	0.52	(0.28, 0.99)	0.04*

*p<0.05

^a^ Controlled for sex, age, BMI, and region

†p<0.01, OR: Odds Ratio, CI: Confidence Interval, Ref: Reference category, PE: Physical education, ECA: Extracurricular activities, PA: Physical activity.

Results of univariable regressions of school policies and practices related to students’ active play during four free periods at schools are shown in Table 4. Although other policies related to PA promotion (OR = 0.49, 95%CI: 0.31–0.78, p = 0.002) and having a PE specialist at school (OR = 0.54, 95%CI: 0.38–0.76, p<0.001) seemed to have an inverse association with students’ active play of ≥2 free periods at school in the univariable regressions, no factors were significantly associated with students’ active play when accounted for potential confounders and other variables in the multivariable regressions.

**Table 4:** Univariable regression results of school variables associated with students’ participation in active play^†^.

	% Participation in active play		OR	(95%CI)	p
	No (Ref)	Yes			
**Policy**					
Recess	20.4	14.6	0.66	(0.43, 1.01)	0.058
PA facilities/equipment	20.9	17.7	0.82	(0.40, 1.68)	0.595
Involvement in HPS	14.4	18.6	1.35	(0.74, 2.47)	0.327
Active play	18.3	17.6	0.95	(0.64, 1.41)	0.812
Other PA-related policies	29.3	17.1	0.49	(0.31, 0.78)	0.002*
**Practice**					
Having PE specialists	26.4	16.2	0.54	(0.38, 0.76)	<0.001
Organization of ECA	16.8	17.9	1.06	(0.54, 2.10)	0.859
Student access to indoor PA facilities outside school hours	21.5	17.2	0.75	(0.48, 1.20)	0.241
Student access to outdoor PA facilities outside school hours	25.0	17.3	0.62	(0.38, 1.02)	0.061
Community access to indoor PA facilities outside school hours	17.8	17.9	1.01	(0.68, 1.50)	0.975
Community access to outdoor PA facilities outside school hours	18.5	17.6	0.94	(0.57, 1.55)	0.807
**Characteristic**	**City district (Ref)**	**Non-city district**			
Setting	18.2	17.3	0.94	(0.61, 1.43)	0.776

*p<0.05, OR: Odds Ratio, CI: Confidence Interval, Ref: Reference category, PA: Physical activity, HPS: Health promoting school, PE: Physical education.

^†^Results of multivariable regressions are not shown as no significant associations were found between explanatory and outcome variables.

Students’ participation in active transport to/from school was significantly associated with the existence of a school active transport policy in both univariable and multivariable regressions. When accounting for potential confounders and other factors in the model, students were 40% (OR = 1.40, 95%CI: 1.09–1.82 p = 0.01) more likely to travel actively, compared with their counterparts from schools without this policy, as seen in Table 5.

**Table 5:** Regression results of school variables associated with students’ active travel to/from school.

	% Participation in active travel		Univariable regression			Multivariable regression^a^		
	No (Ref)	Yes	OR	(95%CI)	p	OR	(95%CI)	p
**Policy**								
Involvement in HPS	60.7	50.9	0.67	(0.40, 1.12)	0.132			
Active transport	49.0	56.9	1.37	(1.00, 1.87)	0.043*	1.40	(1.09, 1.82)	0.01*
Other PA-related policies	48.0	52.9	1.21	(0.78, 1.88)	0.382			
**Practice**								
Having PE specialists	54.5	52.2	0.91	(0.60, 1.38)	0.665			
**Characteristics**								
Setting	**City district (Ref)**	**Non-city district**						
	51.4	54.5	1.13	(0.81, 1.57)	0.460			
Neighborhood issues	**Low (Ref)**	**High**						
	52.7	52.3	0.98	(0.59, 1.63)	0.951			

*p<0.05

^a^ Controlled for sex, age, BMI, and region, OR: Odds Ratio, CI: Confidence Interval, Ref: Reference category, PA: Physical activity, HPS: Health promoting school, PE: Physical education.

## Discussion

This present study revealed some interesting results. Contrasted with low PA levels among Thai children, a high prevalence (>80%) of Thai schools reported a policy promoting PA in many different areas (e.g., PE, community shared-use of school PA facilities, and HPS). Also, a very high prevalence (>90%) of schools reported practices supporting policies and PA promotion (i.e. provision of annual fitness test, organization of extracurricular activities, and student access to PA facilities outside school hours). Nevertheless, almost none of the school PA policies, practices, and characteristics examined in this present study was associated with students’ overall and domain specific activities, except the policy promoting active transport to/from school. The results suggested that school PA policy might be ineffective in influencing overall PA amongst Thai children, or that school policies might not be constructed to support PA, or their implementation might not be translated into supportive actions.

Counterintuitively, our study showed that no school PA policies, practices, and characteristics had an association with students’ overall PA levels. The results contrasted with some previous studies which concluded that a policy promoting active play [31], recess [7], extracurricular activities [6,8,32], and general health through the HPS framework [33] was positively related to students’ overall PA. Additionally, other studies also showed a positive relationship between students’ total PA and multiple school PA policies (measured by a composite index) [34,35], and allowed student access to PA facilities outside school hours [36]. Nevertheless, our findings are consistent with some studies which demonstrated that school policies (measured by a composite index) were unrelated to students’ self-reported [37] or accelerometry-assessed PA [38]. Beets and colleagues found provocative results in their study as written school policy (promoting PA in general) had an inverse association with students’ PA [39]. These inconsistent findings might be affected by different measures used to assess policy, for example, some used a single policy question and some used a composite index. A standardized policy measurement will need to be developed and agreed among researchers in this field to help better clarify the influence of school policies on student’s PA.

PE policy is believed to contribute greatly to children’s PA [13], but the results from our study showed that PE policy had no association with students’ PA levels. Similar findings concluded that PE policy or practices in schools might not contribute to students’ overall PA [6,37,40]. In our case, school policy and practices concerning PE are unlikely to be major factors influencing students’ PA, due to the high level of PE offered by schools and high level of participation recalled by students across all areas. However, unlike Thailand, most previous studies did not observe high levels of PE delivery by schools and high levels of attendance by students, and this raises important questions for future research. Firstly, on the level of implementation of PE policies including quality and fidelity of PE class delivery in Thailand. Secondly, on the potential overall contributions that PE can make to students’ PA levels with one class of <60 min/week, and whether it is better to offer more PE.

While general PA policies may have limited impact on overall PA, some specific policies, such as those related to active transport were powerful in influencing students’ domain specific PA behavior. The school active transport policy showed a positive association with students’ participation in active travel to/from school, although the actual contributions of school active travel to overall PA among Thai children is unknown. Nonetheless, the relationship identified in our study was consistent with a previous Czech Republic study [21], where students made active travel more frequently when their schools had a policy promoting walking and cycling to/from school. The school active transport policy in Thailand, like many other countries, is not mandated by any government authorities. This policy is usually developed at the school level through the school executive board and any policies developed from schools’ own interests might reflect a strong commitment to its implementation, and potentially the alignment of necessary resources required to achieve the desirable outcome behavior [20,21]. Unfortunately, details of the development, specific strategies and implementation of school active transport policies were not gathered as part of this study. The results however have important implications, as extensive evidence shows that students’ participation in active travel to/from school significantly contributes to their greater overall PA [41]. This present study suggests that specific policies targeting specific PA behavior like the school active transport policy is beneficial and could bring about desirable outcome behavior. Further research on the development of school active transport policy including details of implementation is needed.

Conversely, a policy specifically promoting active play at school had no relationship with students’ PA during school free periods, although this policy is initiated at the school level, similar to the active transport policy. Policies concerning recess frequency or duration and provision of PA facilities and equipment including relevant practices, which seemed to be related to students’ activities during school free time, neither showed an association. These findings were consistent with a Canadian study [42]. However, our results conflicted with those of Ridgers and colleagues who found that written policy (promoting PA in general) had a positive association with PA during recess periods [7]. The lack of an association in this present study might be due to several reasons. First, children’ active play was a less obvious outcome behavior and measured indirectly through opportunities during school free periods e.g. recess, and lunchtime. To date, there are no valid and reliable measurement methodologies and instruments to accurately measure children’s active play (Tremblay et al., 2016). Second, the active play policy might actually have a limited effect on students’ choices for their activities during school free time. Multiple factors, apart from school policy, e.g., students’ preference for play space, safety concerns, and recreational environment, may have stronger influence on students’ engagement in active play (Lee et al., 2015). Finally, the implementation of this policy might play a significant role. Details of the implementation, for instance availability of guidance for implementation, school surveillance or supervision during school free periods, and school physical environmental support towards children’ active play would be helpful in explaining the lack of an association. Future studies need to unfold this missing information and studies with a robust measurement of active play are definitely warranted to clarify actual contributions of the policy promoting active play on children’ activities during school free time.

In light of students’ participation in PE class, other PA-related policies showed a positive association to this domain specific activity. It is possible that additional exposure to PA, other than PE class, through other PA-related policies, might indirectly reflect the importance of PE and lead to increased participation in PE class [43]. However, it is still questionable in the current study as the details of other PA-related policies adopted by schools were lacking. Further investigation is necessary in order to understand the identified positive relationship.

The key strengths of this study include the use of a previously validated instrument to collect Thai students’ PA data, and the use of nationally representative data of children and youth in a wide age spectrum of 6–17 years across the country. Despite the low response rate to the principals’ survey of 44.3%, this study has provided some baseline Thai data on school PA policies and practices. Though there is a potential selection bias through the principals’ response rate, a comparison between students’ participation in overall PA, PE, active play and transport revealed no significant differences between participating and non-participating schools in the principal survey. Data on school policies, practices, and characteristics including students’ participation in PA were based on self-reported measures. The school data might have been biased due to school principals’ social desirability of supporting students with opportunities for PA in school. Data on implementation of the policies reported which would allow more understanding of their influence on students’ participation in PA remain largely unknown. Additionally, the very high proportion of schools with PA-related policies made it difficult to compare between schools and student behaviors, and therefore, the overall influence of policies on student behaviors. Finally, a causal relationship between explanatory and outcome variables cannot be established from this cross-sectional study.

## Conclusions

This study provides an important new finding on the contributions of school PA policies to children’s overall and domain specific PA. The finding supports the importance of establishing a formal policy position that should be specific to a targeted PA behavior. Despite a high prevalence of Thai schools reporting many policies and practices promoting PA in different areas, students reported low levels of overall PA. This study suggested that none of the school PA policies, practices, and characteristics was associated with students’ overall and domain specific activities, with active transport as the one exception. The policy promoting active transport to/from school seemed to positively influence students’ engagement in active travel. While this policy may need updating in the current situation, it remains a significant finding and helps underscore support for wider adoption of the policy promoting active transport to/from school which is designed for a specific outcome behavior amongst Thai schools. Intervention studies are necessary to confirm this finding. Our study also reflects that, at this stage, policy formulation might not be an issue among Thai schools. Concentration on strategies to translate the existing policies into effective implementation will be a priority, if greater impacts of PA policies are to be expected.
